# Effects of extracorporeal shockwave therapy versus ultrasonic therapy and deep friction massage in the management of lateral epicondylitis: a randomized clinical trial

**DOI:** 10.1038/s41598-024-67313-1

**Published:** 2024-07-17

**Authors:** Wajida Perveen, Sahreen Anwar, Riaz Hashmi, Misbah Amanat Ali, Asim Raza, Umer Ilyas, Shibili Nuhmani, Masood Khan, Ahmad H. Alghadir

**Affiliations:** 1 CMH Lahore Medical College & IOD (NUMS Rawalpindi), Lahore Cantt, 54810 Pakistan; 2 Faculty of Rehabilitation Sciences, Lahore University of Biological and Applied Science, Lahore, Pakistan; 3 Department of Physical Therapy, Syed Medical Complex, Sialkot, Pakistan; 4 Department of Physical Therapy, Avicenna Medical College, Lahore, Pakistan; 5 Department of Physical Therapy, College of Applied Medical Sciences, Imam Abdulrahman Bin Faisal University, Dammam, Saudi Arabia; 6 Rehabilitation Research Chair, Department of Rehabilitation Sciences, College of Applied Medical Sciences, King Saud University, Riyadh, Saudi Arabia

**Keywords:** Tennis elbow, Pain, Physiotherapy, Patient-rated tennis elbow evaluation questionnaire, Musculoskeletal system, Rehabilitation

## Abstract

The study's goal was to compare and evaluate the benefits of deep friction massage and ultrasonic therapy (US) vs extracorporeal shockwave therapy (ESWT) for people with lateral epicondylitis. This double-blind, parallel-arm randomized clinical trial was conducted after ethical approval on a sample of 80 subjects with lateral epicondylitis. Participants were enrolled based on predefined eligibility criteria. They were randomly allocated to groups A and B. Group A received ESWT, while Group B received the US combined with deep friction massage. Data was collected using the Numeric Pain Rating Score (NPRS) and Patient-rated tennis elbow evaluation questionnaire (PRTEE) at baseline, at 3rd, and at 7th week of treatment. On the basis of the normality of the data, a non-parametric test was applied to evaluate between-group and within-group differences. P value ≤ 0.05 was considered significant. There was a significant difference between groups (p < 0.001). Comparisons of PRTEE scores at 3rd week and 7th week of intervention were found significant for both groups (p < 0.001). While considering between-group comparisons based on percentile scores of PRTEE at baseline, 3rd and 7th week of intervention, in group A Median (IQR) at the baseline was 24.00 (5.00), at 3rd week, 10.00 (5.00) and 7th week was 1.50 (2.50) and in group B Median (IQR) at the baseline was 25.00 (4.00), at 3rd week 19.50 (4.50) and at 7th week was 11.50 (2.50). The results were significant in both groups (p = 0.000), but between-group analysis revealed that ESWT is more effective in patients with lateral epicondylitis.

## Introduction

Lateral epicondylitis is one of the common ailments in the elbow region, affecting 1–3% of the adult population every year^1^. The common presentation of epicondylitis is in the form of elbow pain caused by inflammation or degeneration of the Common extensor tendon, usually due to repeated activity. Female gender, smoking history, repeated activity and ipsilateral scapulothoracic dysfunction are common associated factors^2,3^. The clinical examination, functional impairment, and positive provocation tests like Cozen's test and Mills's test form the basis of diagnostic criteria for lateral epicondylitis^4^. Musculoskeletal ultrasound is one of the gold standards for detection of Lateral epicondylitis^5^.

Patients suffering from lateral epicondylitis are treated with many interventions ranging from conservative to surgical^6^. Most preferred interventions include iontophoresis, friction massage, electrotherapy, strengthening exercises and corticosteroid injections. These treatment strategies show long-term results, whereas oral medications show short-term results^7^. Ultrasound therapy and friction massages are routinely used to treat the swollen tendon and underlying adhesions. The thermal effects caused by ultrasound boost metabolic activity in the area, improving blood flow, whereas its nonthermal effects include collagen synthesis and tissue repair^8^. Transverse friction massage is used to break adhesions, increase blood flow and reduce inflammation in chronic tendinopathies^9^.

There is a recent surge in the use of Extracorporeal shock wave therapy (ESWT) in the treatment of tendinopathies such as lateral epicondylitis. Shockwave therapy (SWT) is believed to stimulate tissue repair and regeneration in chronic tendinopathies like lateral epicondylitis (LE) through several mechanisms like neovascularization, cell proliferation and anti-inflammatory effects^10^. The healing effects of ESWT are due to mechano-transduction, a process by which the body alters mechanical load into cellular one, which ultimately promotes structural change^11^. Both ESWT and ultrasonic therapy (US) show good results in the treatment of lateral epicondylitis by alleviating pain through increased blood flow to the area causing hyperstimulation analgesia^12^.

Evidence suggests that pain and functional abilities are improved in patients undergoing ESWT or ultrasound therapy; however, the most effective treatment strategy for the treatment of lateral epicondylitis is not well known, and there is a need to address optimal treatment parameters, long-term outcomes, comparative cost-effectiveness, and comprehensive patient-reported outcomes^13^.

This study aimed to compare the therapeutic effects of ESWT alone versus US and deep friction massage on pain relief and functional ability in patients diagnosed with lateral epicondylitis. The study hypothesized that there would be a significant difference between the therapeutic effects of ESWT alone and US with deep friction massage.

## Materials and methods

### Study design and settings

This parallel arm randomized clinical trial, conducted between December 2020 and December 2021, took place at two locations: the Physiotherapy Department of Syed Medical Complex, Sialkot, and the Physiotherapy Department of Sialkot College of Physical Therapy at Amin Welfare & Teaching Hospital, Sialkot. The trial followed a double-blind design, with both the patients and outcome assessors being unaware of the treatment assignments. Written informed consent was obtained from all participants in accordance with the principles outlined in the Declaration of Helsinki. The study received ethical approval from the ethics committee of Sialkot College of Physical Therapy CPT-IRB, with reference number IRB-SCPT-DPT-140-2020. Prior to enrolling the first participant, the trial was prospectively registered in the Iranian Registry of Clinical Trials (IRCT) with the registration number IRCT20201031049207N4 (date of registration 25/12/2020). The study adhered to the CONSORT guidelines for reporting clinical trials, and all procedures were performed in compliance with the relevant guidelines.

### Sample size calculation

The sample size of 74 patients was calculated through the Giga Calculator with 95% confidence level and 80% power of the study. By adding a 10% attrition rate, the sample size was 80. The sample size was estimated based on the mean and standard deviation of pain scores in a study by Staples et al.^14^.

### Participants, blinding, and eligibility criteria

Health conditions studied were lateral epicondylitis or Tennis Elbow ICD-10 code M77.10. Participants were recruited from a multicentre, and an initial screening form was filled out. A blind assessor explained the whole procedure and the right to draw from the research at any time. Male and female patients aged between 30 and 60 years, localised pain for more than eight weeks, and with positive Mill's and Cozen's tests were included in the study^15^. Patients with a history of trauma, surgery, prostheses in the elbow area, sensory deficit and those with use of analgesics in the last three weeks or on corticosteroid injections were excluded from the study.

### Randomization and masking

Patients were divided into two groups randomly through the sealed envelope method. To ensure the concealment of allocation, a method known as sequentially numbered, opaque, sealed envelopes (SNOSE) was employed, following the guidelines outlined by Doig and Simpson^16^. The procedure involved an independent researcher who was not involved in the clinical aspects of the trial and prepared eighty opaque envelopes. Each envelope contained a folded paper indicating the assigned treatment, with an equal distribution of treatments. Randomized numbers were assigned to these envelopes, and they were thoroughly shuffled before being arranged in sequential order. The envelopes were then handed over to another independent researcher. The therapist recorded relevant information on each envelope, which was subsequently opened to reveal the assigned treatment, maintaining concealment. The assessor documented the post-treatment findings, and an additional independent analyst was responsible for data analysis. In this study, both the patients and the assessor were blinded to the allocation of treatment groups.

### Intervention Group A

**Figure 1:**
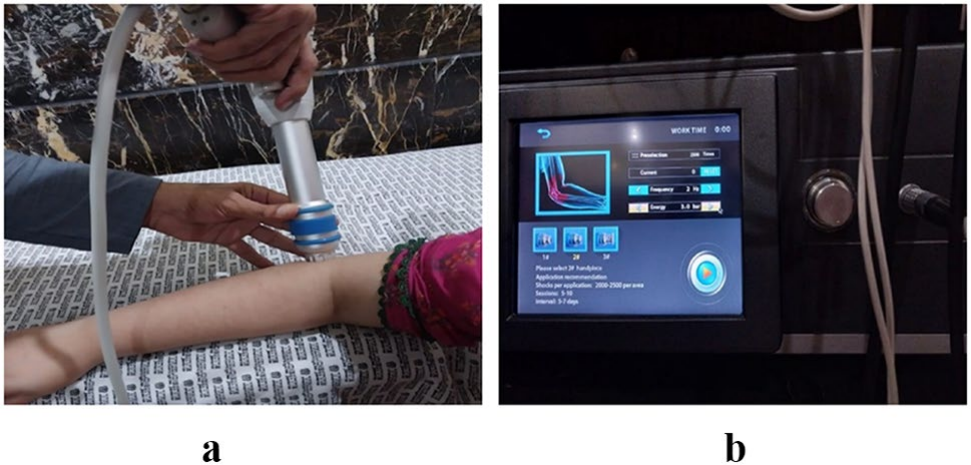
(**a**) Application of extracorporeal shockwave therapy on the right elbow of a patient with lateral epicondylitis. (**b**) Output settings of the extracorporeal shockwave therapy device for a patient with lateral epicondylitis.

In group A, patients received extracorporeal shock wave therapy. The patient was seated with their elbow flexed and supported at the edge of the couch. ESWT with decompression (ESWT-Japan, Model JESL-2000 Shockwave Therapy System) was administered on the lateral side of the elbow, utilizing a frequency of 1 Hz, an energy level of 2.0, and a pressure bar of 2 as shown in Fig. 1a and b^17^. Each treatment session lasted for 5 min, and a total of 7 sessions were conducted with a 4-day interval between each session. Evaluations were performed at Week 0, the 3rd week, and the 7th week of the treatment for all participants.

### Intervention Group B

**Figure 2:**
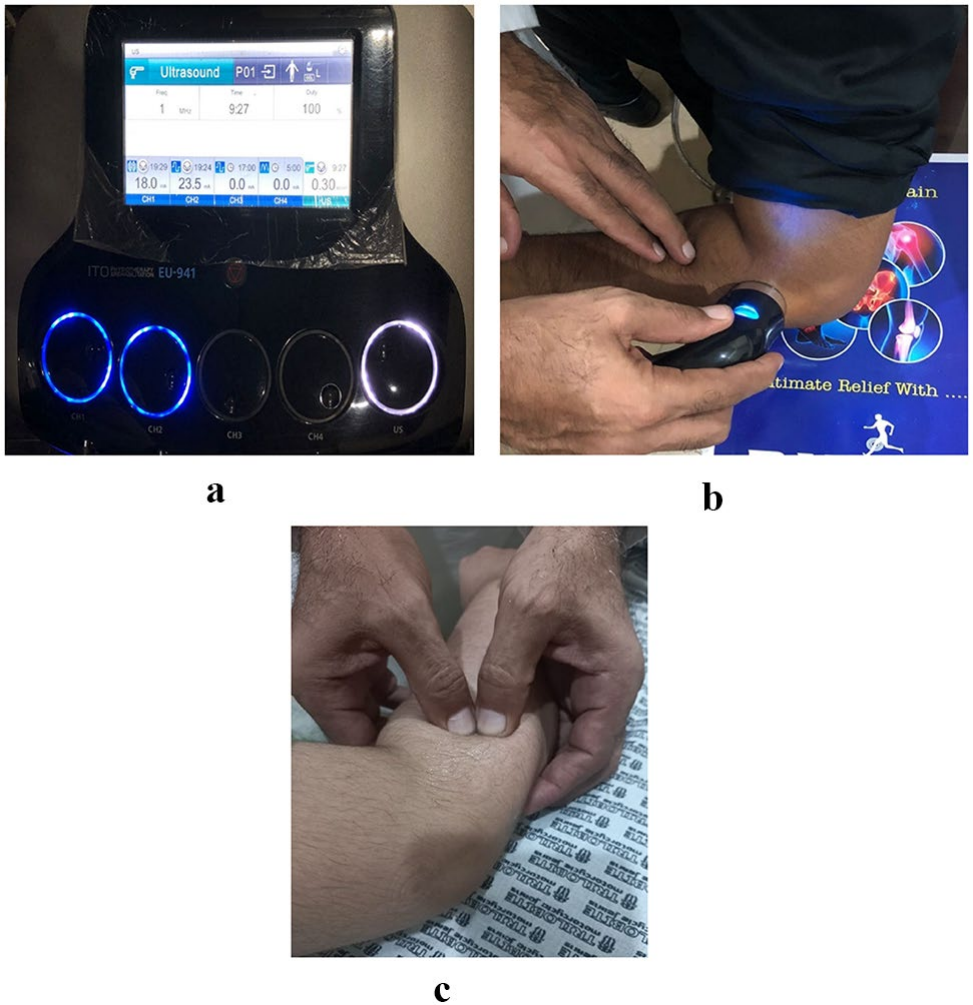
(**a**) Output settings of the ultrasonic therapy device for a patient with lateral epicondylitis. (**b**) Application of the ultrasonic therapy for a patient with lateral epicondylitis. (**c**) Application of the deep tissue friction massage for a patient with lateral epicondylitis.

In group B, patients received a combination of US and deep friction massage. The patient assumed a seated position with the elbow flexed and supported at the end of the couch. The US was administered through ITO-EU 941 (Japan) multi-frequency treatment head at a rate of 2.5w/sec2 for 3–5 min, using an intermittent mode, three times per week for a total of seven weeks, as shown in Fig. 2a and b^18^. The Deep friction massage was used as an adjunct to the US for 2–5 min, as shown in Fig. 2c. The massage was applied directly to the common extensor tendon at the lateral elbow in a perpendicular direction to the orientation of the tendon fibres. The pad of the thumb was used to apply firm, deep pressure in a transverse friction motion across the tendon. The intensity of the pressure was estimated to be 2.3 kg/cm^2^. All the patients were evaluated at baseline, 3rd and at 7th week of the treatment^19^**.**

### Outcome measures

The clinical and functional outcomes of all patients were assessed at the beginning of the treatment, as well as at the 3rd and 7th weeks, using two measurement tools: the NPRS score and the Patient Rated Tennis Elbow Evaluation Questionnaire (PRTEE) score. The PRTEE is a specialized assessment tool designed for individuals with lateral epicondylitis. It comprises 15 items and is divided into three subgroups: pain, special activities, and daily living activities. Higher scores on the PRTEE indicate greater levels of pain and functional impairment, with a score of 0 indicating no disability. Numeric Pain Rating Scale (NPRS) was used to assess pain intensity on a scale of 0 to 10, having a moderate level of reliability with an ICC value of 0.67, indicating consistent and stable scores obtained from the measurement tool^20,21^.

**Figure 3:**
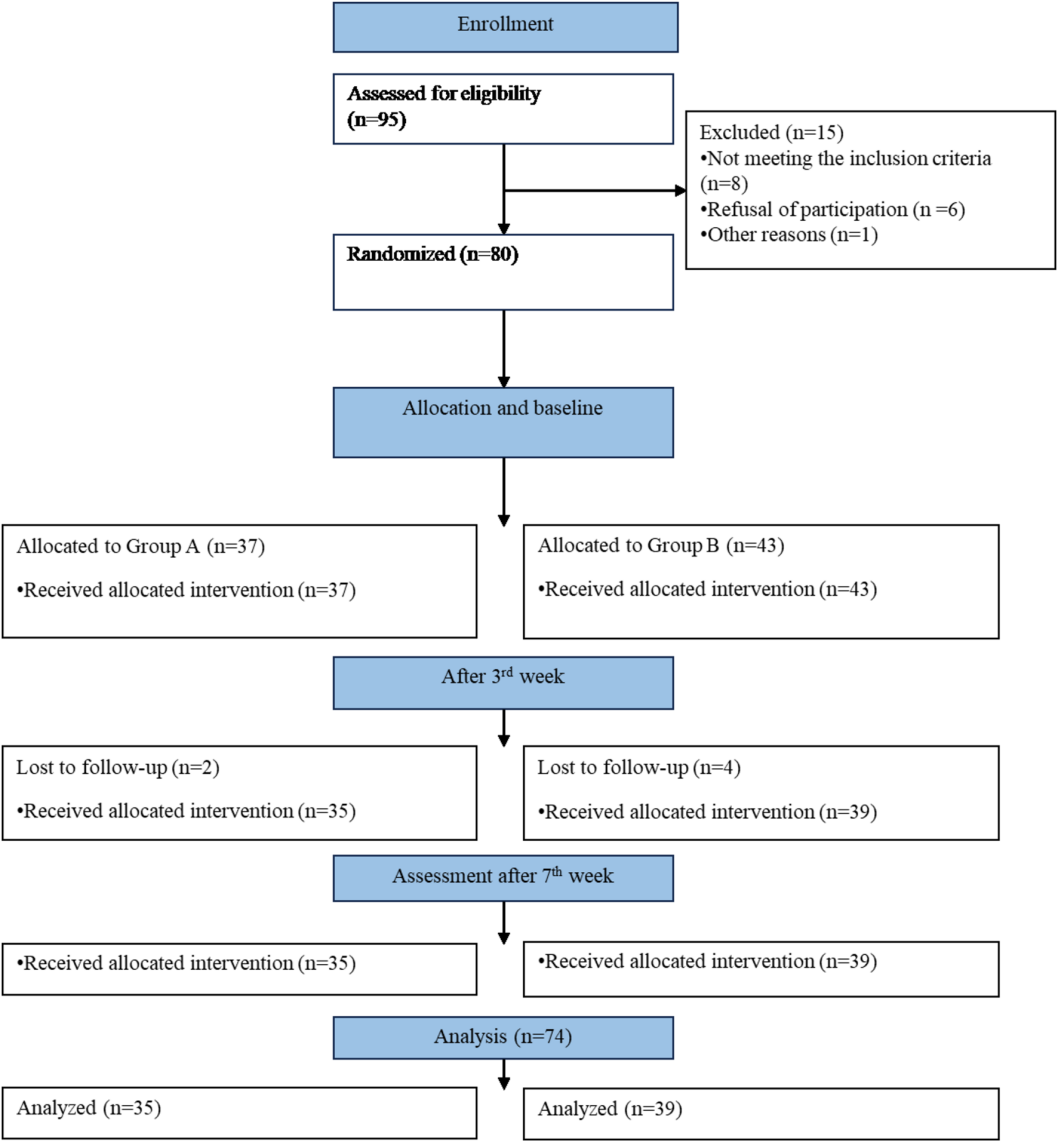
The Consolidated Standards of Reporting Trials (CONSORT) flow diagram shows the recruitment, randomization, allocation, and analysis of participants.

Participant enrolment, randomization, and analysis details are given in the CONSORT flow diagram in Fig. 3. Patients with confirmed diagnoses of lateral epicondylitis visiting the Outpatient Physical Therapy departments of Syed Medical Complex and Sialkot College of Physical Therapy were screened for eligibility. Eligible patients were invited to participate in the study. The willing participants signed written informed consent. They were randomly divided into two groups using SNOOS. Initially, 95 patients were screened over, and 80 were included in the study to meet the sample of 74 subjects, eliminating the attrition. There came unequal distribution in two groups (Group A = 37 and Group B = 43). Treatment was continued as per protocol, and the data was taken at prescribed time points: at baseline, after the 3rd visit, and after the 7th visit in both groups using PRTEE. After 2nd visit, participants were lost in both groups: Group A n = 2, Group B n = 4. Therefore, the study was completed on 74 participants (group A = 35, group B = 39).

### Data analysis

The normality of data was checked using the Shapiro–Wilk's test. The qualitative variables were presented in frequencies and percentages, whereas mean and standard deviation were used to present the mean difference between the quantitative variables. Since the data was not normally distributed, the non-parametric Mann–Whitney U test was used to compare the mean scores between Group A (ESWT) and Group B (control group), while chi-square was used to compare qualitative variables. Likewise, for the analysis of three mean ranks at baseline, after the 3rd and the 7th week, the Friedman test was employed and expressed as Mean (IQR). The statistical significance level in this study was P < 0.05. Data analysis was performed using the Statistical Package for Social Sciences (SPSS) software version 26.

## Results

The mean age ± SD of the participants was 47.58 ± 8.017 years. Baseline and demographic characteristics are given in Table 1. As the data was not normally distributed, a non-parametric test was applied to compute the results. A between-group comparison of the scores PRTEE at baseline, in the 3rd week and the 7th week of treatment based on the Mann–Whitney U test is given in Table 2, while based on percentile scores, it is given in Table 4. Changes in outcomes within groups based on mean rank are expressed in Table 3.

**Table 1:** Baseline and Demographic characteristics of both groups.

Demographic variables	Overall	Group-A	Group-B
Sample size	n = 74	n = 35 (47.3%)	n = 39 (52.7%)
Age (mean ± SD) years	47.58 ± 8.017	47.83 ± 9.096	47.36 ± 7.024
Affected side (elbows)			
Right	30 (40.54%)	16 (45.71%)	15 (38.46%)
Left	36 (48.64%)	15 (42.85%)	20 (51.28%)
Both (2 samples in each)	4 (10.81%)	2 (0.57%)	2 (0.51%)
Gender			
Male	22 (29.7%)	11 (31.42%)	11 (28.20%)
Females	52 (70.3%)	24 (68.57%)	28 (71.80%)
NPRS (mean ± SD)	6.30 ± 3.063	6.75 ± 7.612	7.45 ± 5.463
Duration of symptoms (in average weeks)	26	28	24

*NPRS: Numeric Pain Rating Scale.

**Table 2:** Between-group comparisons of PRTEE scores at baseline, after 3rd and 7th week of treatment.

Outcome at time points	Groups	n	Mean	Std. Dev	Mean Ranks	Mann–Whitney U	Z	P-value
PRTEE at baseline	A	37	25.857	3.9771	36.64	652.5	− 0.326	0.745
	B	43	25.897	3.635	38.27			
PRTEE at 3rd week	A	35	11.143	4.0757	19.67	58.5	− 6.761	< 0.001*
	B	39	21.013	4.0401	53.5			
PRTEE at 7th week	A	35	2.014	2.4479	18	0.000	− 7.442	< 0.001*
	B	39	11.833	2.2634	55			

*Significant.

PTREE: patient-rated tennis elbow evaluation questionnaire.

**Table 3:** Within-group comparison of PRTEE scores at baseline, 3rd week and 7th week of treatment.

Treatment groups	Assessments time points	Mean rank	P value
Group-A	PRTEE at baseline	3.00	0.000*
	PRTEE at 3rd week	2.00	
	PRTEE at 7th week	1.00	
Group-B	PRTEE at baseline	2.99	0.000*
	PRTEE at 3rd week	2.01	
	PRTEE at 7th week	1.00	

*Significant.

PTREE: patient-rated tennis elbow evaluation questionnaire.

Table 1 shows that there were 74 participants in total with mean age ± SD 47.58 ± 8.017 years, while 35 participants in group A (ESWT) with mean age ± SD 47.83 ± 9.096 years, and 39 participants in group B (US with deep friction massage) with 47.36 ± 7.024 years. Affected elbows were 30 (40.54%) on the right, 36 (48.64%) on the left side and 4 (10.81%) with bilateral elbows. In total, 22 (29.7%) were male, and 52 (70.3%) were females. Overall NPRS was 6.30 ± 3.063, with 6.75 ± 7.612 in group A and 7.45 ± 5.463 in group B. The average duration of onset of symptoms was 26 weeks.

Table 2 shows between-group comparisons of PRTEE scores at baseline, at the 3rd week and 7th week of intervention and was found significant at the 3rd and 7th week post-intervention (P < 0.001).

Table 3 shows a steady improvement within both groups A and B (P = 0.000). However, it is more consistent in group A, with a mean rank of 3.00 at baseline, 2.00 after 3rd week and 1.00 after 7th week of intervention in contrast with the mean rank in group B mean rank 2.99 at baseline, 2.01 at 3rd week and 1.00 at 7th week of intervention (Table 4).

**Table 4:** Between-group comparisons of PRTEE scores at baseline, 3rd week and 7th week of treatment based on percentile scores.

Groups	Percentiles	Baseline PRTEE	3rd week PRTEE	7th week PRTEE	P value
Group-A Shockwave therapy N = 35	25th	23.00	8.00	0.00	0.000
	50th	24.00	10.00	1.50	
	75th	28.00	13.00	2.50	
Median (IQR)		24.00 (5.00)	10.00 (5.00)	1.50 (2.50)	
Group-B Ultrasonic therapy + deep friction massage N = 39	25th	23.00	18.50	10.00	0.000
	50th	25.00	19.50	11.50	
	75th	27.00	23.00	12.50	
Median (IQR)		25.00 (4.00)	19.50 (4.50)	11.50 (2.50)	

**Figure 4:**
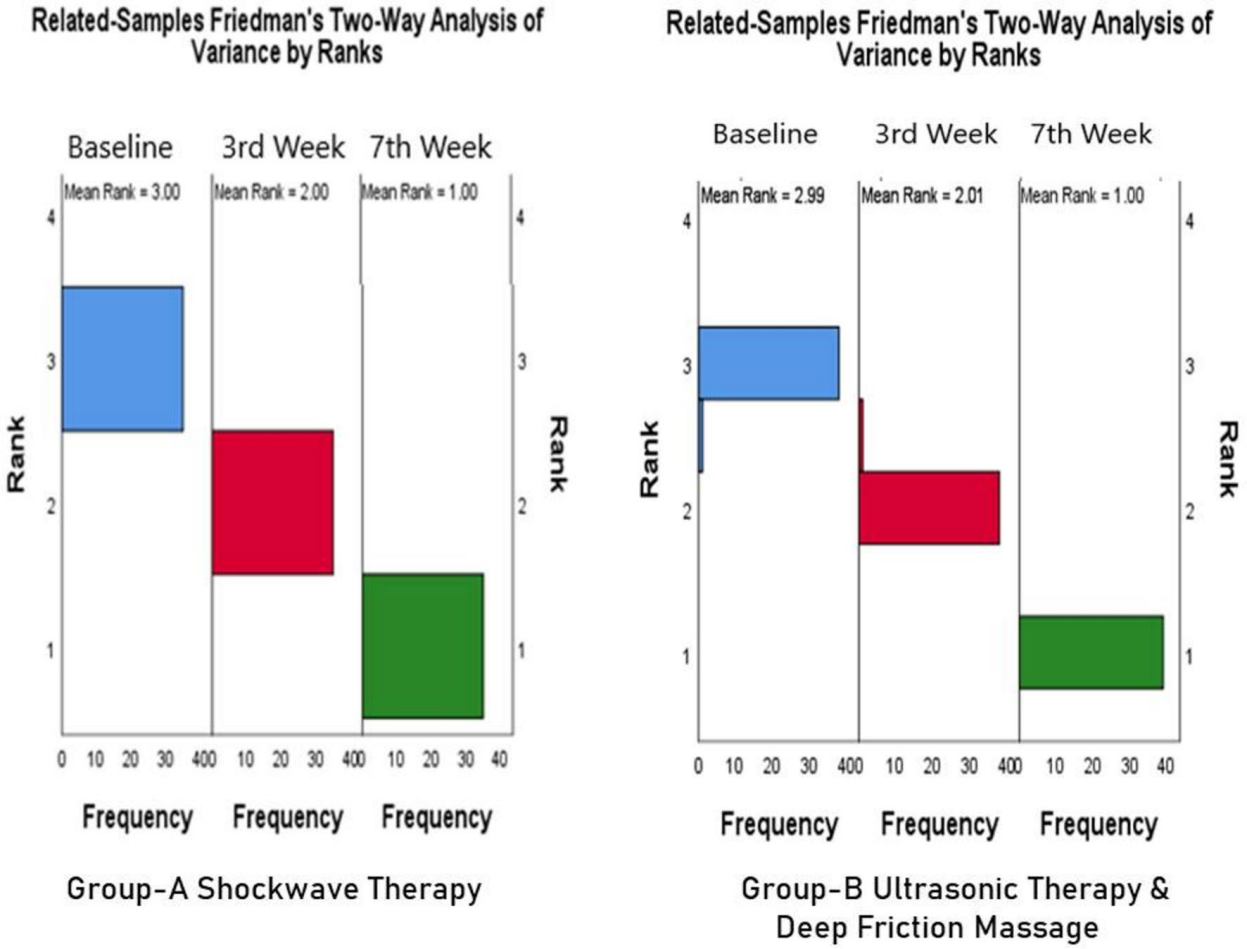
Between-group comparison of mean ranks PTREE Scores at baseline, 3rd week and 7th week.

The table shows between-group comparisons based on percentile scores of PRTEE at baseline, after 3rd, and 7th week of intervention (Fig. 4). In group A, the Median (IQR) at the baseline was 24.00 (5.00), in the 3rd week, 10.00 (5.00) and in the 7th week, it was 1.50 (2.50). In group B, the Median (IQR) at the baseline was 25.00 (4.00), in the 3rd week, 19.50 (4.50), and in the 7th week was 11.50 (2.50). While the results were significant in both the groups (P = 0.000).

## Discussion

Lateral epicondylitis is a chronic elbow joint condition which is mainly manifested as pain and tenderness over the lateral condyle of the elbow^22^. According to previous studies, there are plenty of treatment options, from conservative to surgical, for the treatment of lateral epicondylitis^6^. There is an increasing trend in the use of ESWT and US due to the noninvasive nature of the modalities^23^. This study was conducted to compare the effectiveness of ESWT and US for the treatment of lateral epicondylitis. The results of this study suggested that ESWT was more effective in treating pain and reducing disability as compared to US and deep friction massage.

The results of this study are in accordance with a systematic review in which 13 articles with 1035 participants were included. According to the results, ESWT was effective in reducing pain VAS (p = 0.0004) and improving grip strength (0.0001) caused by lateral epicondylitis. In another study, ESWT showed an 89.6% success rate at 3 months follow-up and a 93.7% success rate at 6 months follow-up in patients who failed the conservative treatment. In addition, at one-year follow-up, there was a 0% recurrence rate in patients who underwent treatment with ESWT^24^.

According to a study by Aldaja et al. ESWT had a superior effect as compared to conventional physical Therapy. In this RCT, forty participants completed the five sessions of the intervention program, and it was concluded that ESWT was effective in improving pain and enhancing upper extremity function^25^. The results of this study advocated the fact that ESWT has good analgesic effects. In our study, ESWT showed superior effects when compared to US.

In another study, the effects of dry needling were compared with ESWT in patients with lateral epicondylitis. The study advocated that the two treatments showed no significant difference at any assessment time point. In long-term follow-up, both treatment groups had significant improvement in pain score, whereas no significant difference was found in grip strength and functional impairment. This emphasizes the fact that this noninvasive process can equally benefit patients for pain relief^26^.

In a randomized clinical trial including acute and chronic cases of lateral epicondylitis, it was concluded that ESWT was equally effective in treating acute and chronic cases of lateral epicondylitis. In addition, it was observed that it prevented the acute cases from developing chronicity. The outcome measures were assessed on the 2nd, 6th and 24th week, a time good enough to observe the effects of ESWT^27^.

The superiority of ESWT over US has been reported in the literature. According to a meta-analysis, ESWT proved to be a more effective treatment than US in reducing pain and improving grip strength in patients with Lateral epicondylitis^23^. When compared to surgical intervention, ESWT is a cost-effective treatment depending on the number of sessions and the duration of treatment. It is feasible in terms of access and its noninvasive nature.

The limitation of the study lies in the fact that it is a small-scale study with limited outcome measurement tools. The addition of more sophisticated tools could add strength to the study. The randomization showed the unequal distribution of the participants in both groups, leaving some errors. Moreover, the baseline mean scores on the NPRS were around 6–7, indicating moderate pain levels. This suggests that many participants began the study with relatively low pain scores. Due to the floor effect, the NPRS may not adequately capture further reductions in pain, as participants have limited room to score lower within the scale. For instance, if a participant's pain level decreases from 6 to 4, it might be a significant improvement, but the scale's ability to reflect this change accurately is constrained by its lower bound. Statistical principles and previous research on pain measurement scales support that such floor effects can limit the sensitivity of the NPRS, potentially underestimating the true effectiveness of the treatment. Alternative measures with a wider range or different sensitivity might provide a more accurate assessment of pain reduction in these cases. Another major limitation is the lack of long-term follow-up beyond 7 weeks to assess sustained treatment effects.

## Conclusion

The study comes up with the conclusion that both the interventions of ESWT and a combination of US and deep friction massage are effective in the management of patients with lateral epicondylitis.

## Data Availability

The data associated with the paper are not publicly available but are available from the corresponding author on reasonable request.
